# Exercise is Medicine Canada workshop training improves physical activity practices of physicians across Canada, independent of initial confidence level

**DOI:** 10.36834/cmej.68376

**Published:** 2020-09-23

**Authors:** Myles W. O’Brien, Chris A. Shields, Kara Solmundson, Jonathon R. Fowles

**Affiliations:** 1 School of Kinesiology, Acadia University, Nova Scotia, Canada; 2 Nova Scotia Health Authority, Nova Scotia, Canada; 3 Division of Kinesiology, Dalhousie University, Nova Scotia, Canada; 4 Faculty of Medicine, Department of Family Practice, University of British Columbia, British Columbia, Canada

## Abstract

**Background:**

Educational workshops help physicians (MDs) include physical activity and exercise (PAE) content in more patient appointments. It is unclear if MDs with varying degrees of confidence discussing PAE with their patients equally benefit from such training. We evaluated whether MDs’ initial confidence affects the impact of an educational PAE workshop.

**Methods:**

MDs (*n =* 63) across Canada completed self-reflection questionnaires initially and 3-months following a PAE workshop. MDs were divided into low-confidence [confidence score (out of 100%): <40%; *n =* 21], medium-confidence (40-60%; *n =* 19) and high-confidence (>60%; *n =* 23).

**Results:**

PAE counselling confidence increased in all groups (relative increase: Low=~40%, Medium=~20%, High=~10%). Training increased the low-confidence group’s knowledge, awareness of guidance/resources and perception of their patients’ interest in lifestyle management (~30% change; all *p <* 0.001). Compared to baseline, a greater proportion (all *p <* 0.001) of MDs reported prescribing exercise at 3-month follow-up in each of the low-confidence (10% to 62%) medium-confidence (16% to 89%) and high-confidence (57% to 87%) groups.

**Conclusion:**

PAE training favorably improved MDs’ confidence, perceived impact of many barriers and the proportion of MDs prescribing exercise, at each level of confidence. An educational workshop particularly assisted MDs with low-confidence (i.e., those who needed it the most) integrate PAE into their practice.

## Abstract (fr)

**Contexte:**

Les ateliers éducatifs aident les médecins (MD) à inclure un contenu sur l’activité physique et l’exercice (PAE) dans un plus grand nombre de consultations auprès de leurs patients. On ne peut établir clairement si les médecins, avec divers degrés de confiance en soi pour discuter de l’activité physique et de l’exercice avec leurs patients, bénéficient tous également d’une telle formation. Nous avons évalué si la confiance en soi initiale du médecin influence les effets d’un atelier éducatif sur l’activité physique et l’exercice.

**Méthodes:**

Des médecins (*n =* 63) de partout au Canada ont rempli des questionnaires d’introspection au départ et trois mois après un atelier sur l’activité physique et l’exercice. Les médecins ont été divisés selon un niveau de confiance faible [score d’auto-efficacité (sur 100 %) : < 40 %; *n =* 21], moyen (40 - 60 %; *n =* 19) et élevé (> 60 %; *n =* 23).

**Résultats:**

L’auto-efficacité de services de consultation en matière d’activité physique et d’exercice a augmenté dans tous les groupes (augmentation relative : faible = ~40 %, moye *n =* ~20 %, élevé = ~10 %). La formation a augmenté les connaissances, la sensibilisation aux conseils/ressources et la perception de l’intérêt de leurs patients dans la gestion du style de vie dans le groupe à faible niveau de confiance (changement d’environ 30 %; tous *p* < 0,001). Par rapport à la valeur initiale, une plus grande proportion (tous *p* < 0,001) des médecins ont indiqué prescrire de l’exercice au suivi de trois mois dans chacun des groupes avec un degré de confiance faible (10 % à 62 %), moyen (16 % à 89 %) et élevé (57 % à 87 %).

**Conclusion:**

La formation sur l’activité physique et l’exercice a amélioré la confiance en soi des médecins, les effets perçus de nombreux obstacles et la proportion de médecins prescrivant de l’exercice, à chaque degré de confiance. Un atelier éducatif a aidé plus particulièrement les médecins avec un faible degré de confiance (c.-à-d., ceux qui en avaient le plus besoin) à intégrer l’activité physique et l’exercice dans leur pratique.

## Introduction

Regular physical activity and exercise (PAE) is associated with numerous health benefits and a reduced risk of developing most chronic conditions.^1^ However, only 17% of Canadians meet Canada’s recommended physical activity guidelines of 150 minutes of weekly moderate-vigorous intensity aerobic physical activity in bouts lasting at least 10 minutes.^2^ Primary healthcare providers, such as physicians (MDs), are a viable medium for promoting PAE to a large proportion of the Canadian population. Patients who receive physical activity prescriptions from their MD increase their physical activity and physical fitness levels while lowering their systolic blood pressure and body mass index.^3^^,^^4^ However, only a small percentage of Canadian MDs (~15%) provide their patients with written exercise prescriptions.^5^^,^^6^ Personal knowledge, billing structure, confidence, time constraints, perceived patient interest and awareness of available resources prevent MDs from providing regular physical activity counselling and prescribing exercise to their patients.^5^^–^^7^ It is well-established that confidence is a strong predictor of human behaviour.^8^ Research from our laboratory ^9^^,^^10^ and others^11^^–^^13^ demonstrates that MDs’ confidence in their ability to perform physical activity counselling and prescribe exercise is a key variable on an individual level, proportionally related to the frequency of PAE content included in their patient-provider appointments and increases with effective educational training.

Exercise is Medicine Canada (EIMC) is a national initiative aimed at increasing the number of healthcare providers assessing, counselling and prescribing PAE as part of routine healthcare visits. EIMC embarked on a national program of educational training workshops that resulted increased MDs’ overall confidence to provide physical activity counselling from ~50% to ~75% and the rates of MDs prescribing exercise from 20% to 74%, at 3-months following the PAE workshop.^10^ Another workshop based MD educational training produced improvements in PAE knowledge immediately following an 8-hr workshop^14^ and also at one-month follow-up after a 3-hr workshop.^15^ Furthermore, a mixed methods study of 12 primary care providers observed that 92% of the MDs interviewed reported a positive change in their approach to providing lifestyle counselling to patients and 58% reported a change in their attitude towards patients needing to change a lifestyle behaviour, 3-6 months following a 2-hour workshop.^16^ The objective of these educational training opportunities was to help providers *begin* or *further* include PAE as part of their regular clinical practice. A criticism of providing educational workshops and doing research on these outcomes is the potential presence of outcome efficacy or response bias, where it may be perceived that only providers interested in the content will attend and are already at a high level of competency. To date, previous workshop-based educational training studies^10^^,^^14^^,^^15^ have primarily included MDs with moderate-high confidence and/or PAE knowledge prior to the intervention. It is unclear whether or not the beneficial effects of educational training are influenced by MDs’ initial confidence. Given the well-documented increases in patients’ physical activity levels when MDs provide them with physical activity advice or prescribe them written exercise prescriptions,^17^^,^^18^ and the notion that MDs who have low confidence in their ability to discuss PAE rarely incorporate PAE content in their patient-provider interactions, it is important to investigate if *all* MDs (from low to high confidence) attending educational training benefit, and to what extent.

The purpose of this study, therefore, was to determine if MDs’ initial confidence in providing physical activity counselling and prescribing exercise influenced the impact of a full-day PAE educational training workshop at 3-month follow-up among Canadian MDs. Specifically, we sought to determine if this workshop was effective at improving PAE perceptions and practices in MD attendees who have low confidence in their ability to include PAE (i.e., those who need the training the most) and if MDs with high confidence benefit similarly from a single-day training workshop.

## Methods

### Participants

Participants were recruited from EIMC workshops delivered across 7 provinces. A total of 168 MDs attended the workshop and 153 MDs completed surveys prior to the workshop (91% participation rate). Of these 153 MDs, 63 completed the follow-up reflection survey 3-months post-workshop (41% retention rate). The physicians (*n =* 63) were distributed as follows: Alberta (*n =* 12), British Columbia (*n =* 3), Manitoba (*n =* 7), Nova Scotia (*n =* 19), Ontario (*n =* 8), Quebec (*n =* 8), Saskatchewan (*n =* 2) and one person did not provide a location. Participants were mainly family physicians (*n =* 58) with some specialist physicians (*n =* 5). Based on their confidence composite score (CCS), MDs were divided into low-confidence (*n =* 21; CCS < 40%), medium-confidence (*n =* 19; CCS = 40-60%) and high-confidence (*n =* 23; CCS > 60%) groups. Some participants (*n =* 46) from our previous study that examined MDs PAE practices in response to the EIMC training workshop^10^ are included in our study. Additional participants (*n =* 17) were added from subsequent workshops to adequately determine the impact of the educational training among MDs across a broad range of confidence levels from low, medium and high. The sample size of each confidence group in our study is similar to the total subject sample in a previous study evaluating the impact of a training workshop with 1-month follow-up on MDs PAE practices (*n =* 25).^15^

### Workshop delivery

The workshops were advertised through respective provincial or health regional bodies and were offered across the country in major centres that showed an interest. MDs attended the workshop of their own interest and were able to receive continuing medical education credits if they completed both the baseline and three-month self-reflection survey. The 7-hour EIMC workshops were provided by 1 exercise physiologist and 1 MD. The interactive workshop covered 5 learning objectives: (*i*) discuss the health benefits and safety of regular exercise with patients, (*ii*) use the exercise vital sign (i.e., time spent engaging in moderate-vigorous physical activity per week), (*iii*) provide basic exercise counselling and prescription for patients as part of the periodic health evaluation, (*iv*) utilize a motivational counselling framework for health behaviour change, and (*v*) understand how to monitor aerobic exercise intensity and perform basic resistance exercises. The focus of the workshops was on delivery of content and there was not a formal recruitment of participants for a research project per se. At the end of the workshop participants were given the opportunity to volunteer their self-reflection questionnaire to EIMC for quality assurance and research evaluation. By providing their questionnaire and voluntarily providing their email address for the three-month follow-up survey, participants consented to de-identified secondary use of their data for research evaluations. This study was approved by the Acadia University and Nova Scotia Health Authority Research Ethics Boards.

### Perception and practice questionnaire

The self-reflection questionnaire was adapted from previous activity prescription questionnaires as described previously.^6^^,^^9^^,^^10^ In brief, the questionnaire included demographics, practice history (e.g., frequency of exercise prescription; <10%, 11%-25%, 26%-50%, 51%-75%, 76%-100%) and confidence (0%-100% using 10% intervals). The construct we measured was self-efficacy, which is a specific form of confidence and refers to MDs’ self-perceived confidence to perform physical activity counselling and prescribe exercise. Barrier impact was collected using an ordinal scale from 1 to 4. A lower impact value indicated a weak impact on their ability to provide physical activity counselling or prescribe exercise. Conversely, a greater impact variable indicated a strong impact. Knowledge was assessed using an ordinal scale (i.e., not at all, slightly, moderately, very, extremely). Questions regarding demographic information, current practice history and barriers were based on published research in diabetes education.^19^^,^^20^ Examples of how questions are worded are provided by O’Brien et al.^6^

### Data and statistical analysis

Descriptive statistics (mean ± standard deviation) were completed on primary variables. A confidence composite score (CCS) was calculated as the mean of all confidence items at each time point for each participant. MDs were stratified into low-confidence (<40% CCS), medium-confidence (40-60% CCS) and high-confidence (>60% CCS) based on their pre-educational workshop CCS. These thresholds are based off of our previous cross-sectional study demonstrating that MDs with previous PAE training had a CCS of ~60% and MDs with no previous training reported an CSS of ~40%.^6^ Pre- and post-workshop confidence and barrier impact variables were compared using a 3 × 2 (Group × Time) repeated measures analysis of variance (ANOVA). To determine whether the *changes* in confidence and barrier impact differed between the groups, we conducted a one-way (Group) ANOVA on the change in confidence and barrier impact variables (i.e., difference between follow-up and baseline). The variances of differences were assessed using Mauchly’s test of Sphericity. Bonferroni correction was used to adjust the p-value for multiple comparisons. The percentage of patients receiving PAE readiness assessments, PAE recommendation and exercise prescription are presented as proportional data (%). Due to the non-continuous measurement of the PAE practice data (<10%, 11%-25%, 26%-50%, etc), non-parametric Wilcoxin-signed rank tests compared the proportion of MDs providing PAE readiness assessments, PAE recommendation and exercise prescription at baseline with follow-up for each confidence group. All statistics were completed in SPSS Version 25.0 (IBM, NY) statistical program. Statistical significance was accepted as *p <* 0.05.

## Results

**Demographics:** The characteristics of the low-confidence (*n =* 21), medium-confidence (*n =* 19) and high-confidence (*n =* 23) groups are presented in Table 1. All groups were similar in age, gender, ethnicity, time spent practicing, patients seen per day and time spent per patient.

**Table 1:** Participant demographics.

	Low Confidence (*n* = 21)	Medium Confidence (*n* = 19)	High Confidence (*n* = 23)
Age (years)	51 ± 9	49 ± 10	51 ± 9
Sex (Male/Female)	12M, 9F	8M, 11F	12M, 11F
Ethnicity (% Caucasian)	85%	84%	86%
Experience >10 years (%)	86%	74%	74%
>20 patients /day (%)	52%	47%	65%
<25 mins /patient (%)	67%	63%	65%

Note: Data presented as mean ± SD or proportional (%). No between group differences were observed. Low, medium and high confidence were defined using confidence composite scores of <40%, 40-60% and >60%, respectively.

### Provider PAE confidence

Each confidence item was different (all p < 0.05) between groups at baseline (High > Medium > Low). At follow-up, low-confidence group’s confidence increased by 40-44% and medium-confidence group by 18-27% to provide PAE information, assess patient’s readiness to begin PAE, answer patient PAE questions, provide advice for special PAE considerations, and make appropriate referrals (see Table 2). Despite having a higher baseline confidence (CSS score: 77 ± 8%), the high-confidence group increased 8%-11% (all, p < 0.05) to provide PAE information, answer patient PAE question and provide advice for special PAE considerations. Although trending, no statistically significant differences were observed in the high-confidence groups’ confidence to assess patient PAE readiness (6%, p = 0.11) and providing a referral (7%, p = 0.09) at follow-up. The relative change in confidence from baseline to follow-up was greater in the low-confidence group than in the high-confidence group (p < 0.001 for all variables; Table 2).

**Table 2:** Changes in confidence from baseline to three-months post-workshop among MDs differing in baseline confidence.

	Baseline	Follow-Up	Relative Change (%)
Provide PAE information			
Low Confidence	33 ± 17	72 ± 13*	40^†#^
Medium Confidence	60 ± 16	78 ± 12*	18
High Confidence	83 ± 16	92 ± 11*	8
Assess patient PAE readiness			
Low Confidence	22 ± 15	63 ± 18*	41^†^
Medium Confidence	41 ± 12	68 ± 12*	27^†^
High Confidence	76 ± 12	83 ± 18	6
Answer patient PAE questions			
Low Confidence	28 ± 13	71 ± 13*	43^†#^
Medium Confidence	58 ± 12	76 ± 11*	18^†^
High Confidence	80 ± 14	88 ± 8*	8
PAE advice for individuals with special considerations			
Low Confidence	21 ± 10	62 ± 13*	41^†#^
Medium Confidence	46 ± 11	69 ± 12*	22
High Confidence	71 ± 12	82 ± 13*	11
Appropriate PAE referral			
Low Confidence	23 ± 19	66 ± 20*	44^†#^
Medium Confidence^a^	49 ± 16	68 ± 18*	20
High Confidence	76 ± 21	83 ± 17	7
Confidence composite score			
Low Confidence	25 ± 10	67 ± 12*	41^†#^
Medium Confidence	51 ± 4	72 ± 10*	21^†^
High Confidence	77 ± 8	86 ± 11*	8

Note: Data presented as means ± SD (%). *p < 0.05 to baseline within each group; †p < 0.05 to relative change in high confidence group; ^#^p < 0.05 to relative change in medium confidence group. ^a^n = 18. At baseline and follow-up, the high confidence group was greater than low confidence and medium confidence groups for all confidence variables (all p < 0.05). At baseline, the medium-confidence group was greater than the lower-confidence group for all confidence variables (all p < 0.05).

### Perceived barrier impact

Initially, the low-confidence group rated their lack of personal knowledge and exercise education in medical school as more impactful than the high-confidence group (both, *p <* 0.002), as shown in Table 3. At follow-up, the low-confidence group rated personal knowledge, patient preference for medication management and lack of guidance/resources for those with chronic conditions as much less impactful (~30%, all *p <* 0.001). Neither perceived patients’ disinterest in exercise or lack of time changed following training in the group of low-confidence MDs (both, *p >* 0.11), but the impact of perceived patient’s disinterest decreased in the medium- and high-confidence groups (both, *p <* 0.02). Both the medium- and high-confidence groups also reported lack of guidance/resources for those with chronic disease and patient’s preference for medication as a less impactful barrier at follow-up (all, *p <* 0.02). Only the high-confidence group perceived lack of time or other lifestyle changes more important as less impactful barriers at follow-up (both, *p <* 0.005). No between-group statistical differences (all, *p >* 0.12) were observed for the absolute change in any barrier impact at follow-up.

**Table 3:** Changes in barrier impact initially compared to three-months post-workshop among MDs differing in baseline confidence.

	Initial	Follow-Up	Change (%)
Patients not interested in exercise			
Low Confidence^a^	2.7 ± 0.8	2.4 ± 0.8	12
Medium Confidence^a^	2.7 ± 0.8	2.2 ± 0.7*	18
High Confidence^b^	3.0 ± 0.8	2.3 ± 0.6*	31
Lack of guidance/resources in exercise for those with chronic disease			
Low Confidence^c^	2.9 ± 1.0	2.2 ± 0.8*	33
Medium Confidence^d^	2.5 ± 0.5	1.9 ± 0.5*	35
High Confidence^a^	2.3 ± 0.7	1.8 ± 0.7*	19
Lack of time			
Low Confidence^c^	2.6 ± 0.8	2.4 ± 0.8	10
Medium Confidence^d^	2.6 ± 0.5	2.3 ± 0.5	11
High Confidence^e^	2.5 ± 0.9	1.9 ± 0.7*	23
Patient prefer medication management			
Low Confidence^a^	2.5 ± 0.8	1.7 ± 0.8*	31
Medium Confidence^f^	2.6 ± 1.0	1.7 ± 0.6*	33
High Confidence^g^	2.4 ± 0.7	1.8 ± 0.8*	27
Other lifestyle changes more important			
Low Confidence^d^	2.0 ± 1.0	1.9 ± 0.9†	3
Medium Confidence^h^	2.1 ± 0.6	1.7 ± 0.6	19
High Confidence^g^	1.8 ± 0.7	1.2 ± 0.4*	32
Personal knowledge			
Low Confidence^a^	2.3 ± 0.8†	1.7 ± 0.7*	28
Medium Confidence^f^	2.0 ± 0.6	1.5 ± 0.5*	25
High Confidence^i^	1.4 ± 0.6	1.3 ± 0.5	13
Lack of exercise education in medical school			
Low Confidence^c^	2.6 ± 0.9†	1.9 ± 0.8*†	27
Medium Confidence^h^	1.7 ± 0.6#	1.5 ± 0.8	9
High Confidence^i^	1.7 ± 0.6	1.3 ± 0.5	26

Note: Data presented as means ± SD (%). *p<0.05 to baseline within group. †p < 0.05 to high-confidence group within time point. #p < 0.05 to medium-confidence group within time point. ^a^n=19; ^b^n = 23; ^c^n = 20; ^d^n = 18; ^e^n = 21; ^f^n = 14; ^g^n = 17; ^h^n = 13; ^i^n = 16. No between group differences (all p > 0.05) were observed for the absolute change in barrier impact. Barrier impact is referring to providers’ perception of their own practice unless specified (e.g., lack of time for provider).

**Figure 1:**
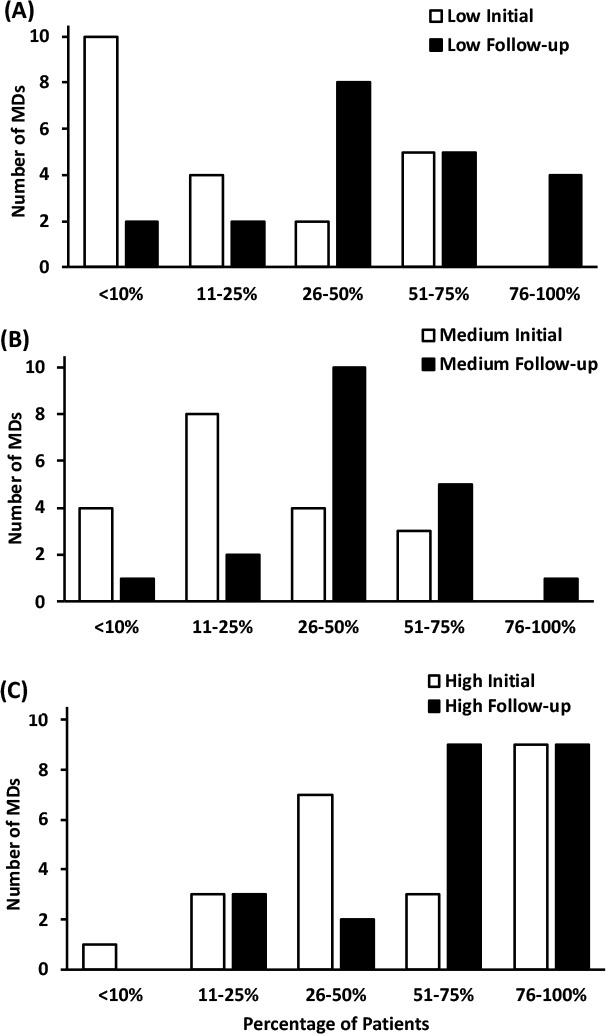
The percentage of patients that physicians report performing PAE readiness assessments on.

**Figure 2:**
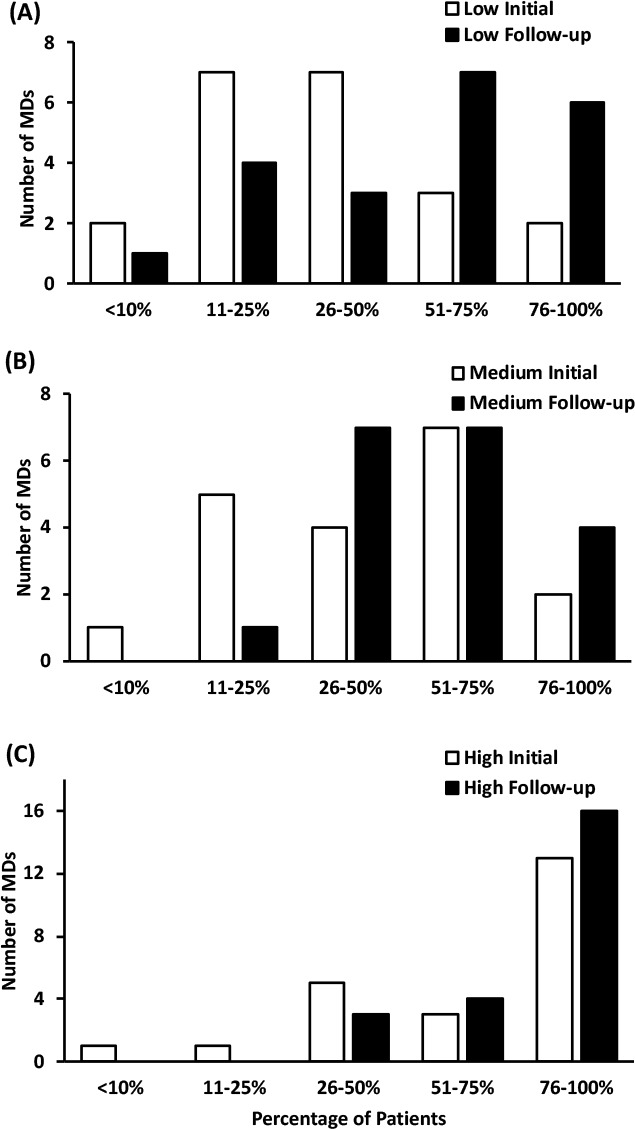
The percentage of patients that physicians report recommending PAE to.

**Figure 3:**
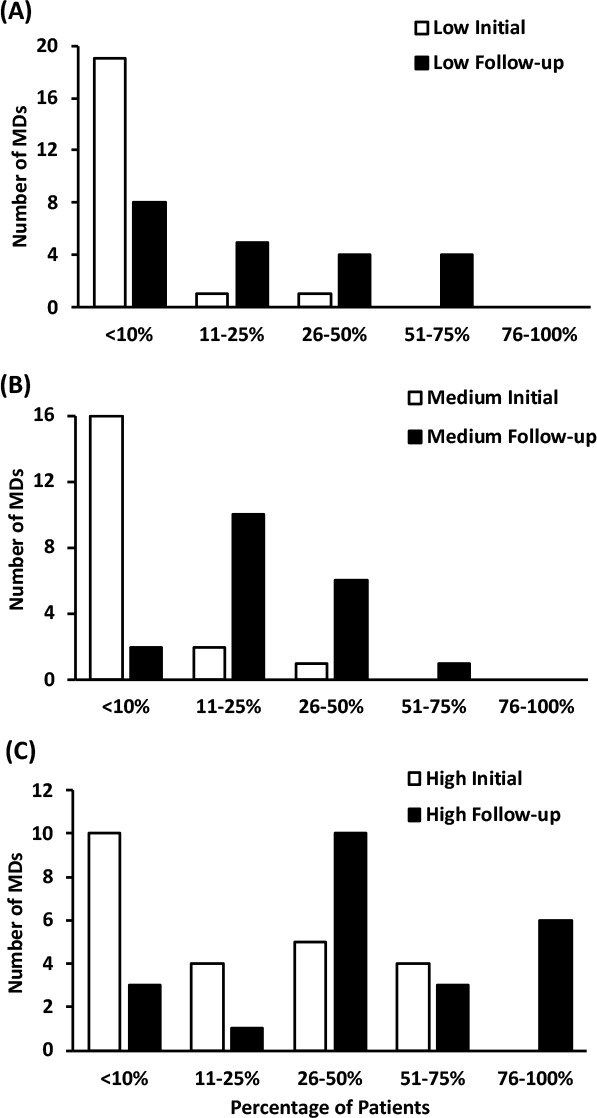
The percentage of patients that physicians report providing written exercise prescriptions to.

**Provider PAE Practices:** Overall, the proportion of MDs who reported assessing PAE readiness increased among low-confidence (*p =* 0.01) and medium-confidence (*p =* 0.03) MDs, but this increase was not statistically significant in the high-confidence group (*p =* 0.058). The percentage of low-confidence MDs assessing patient physical activity readiness most (i.e. >50%) of the time at increased from 24% to 43%, and the proportion of these MDs assessing physical activity readiness in >10% of appointments increased from 52% to 91% (see Figure 1A). The proportion of providers who reported assessing physical activity readiness in most (>50%) patient appointments also increased in the medium-confidence (16% to 32%; Figure 1B) and high-confidence (52% to 78%; Figure 1C) MD groups. Overall, the proportion of MDs who reported recommending PAE increased in all groups (all *p <* 0.05). At follow-up, the percentage of providers recommending PAE to their patients most (>50%) of the time increased among low-confidence (24% to 62%; Figure 2A), medium-confidence (47% to 58%; Figure 2B) and high-confidence (70% to 87%; Figure 2C) groups. Overall, the proportion of MDs who reported providing patients with written exercise prescriptions increased in all groups (all *p <* 0.001). Those who reported prescribing in >10% of patient appointments improved similarly among low-confidence (10% to 62%; Figure 3A), medium-confidence (16% to 89%; Figure 3B) and high-confidence groups (57% to 87%; Figure 3C).

## Discussion

The purpose of this study was to assess if MDs’ initial confidence influences the short-term impact of an EIMC training workshop on physical activity counselling and exercise prescription practices among MDs across Canada. The findings of the present study demonstrate that an EIMC workshop has the capacity to help all MDs; however, those who have low initial confidence (or those who need it the most) drastically increased their confidence (~40%) to perform various aspects of physical activity counselling and helps them begin prescribing exercise. Physicians who were already confident (i.e., CCS: 77±8%) further increased their confidence (~10%), decreased the impact of many perceived barriers and provided even more of their patients with written exercise prescriptions. We demonstrated that educational training designed to assist MDs integrate PAE as part of regular clinical care is effective for all MDs; however, the magnitude of change is different by confidence level and the means by which the training improves clinical practice varies based on initial confidence.

As reported in a recent Canadian Academy of Sport and Exercise Medicine position stand in, patients who received written exercise prescriptions from their MD increased their physical activity level and generated many favorable clinical outcomes such as reduced blood pressure, glycosylated haemoglobin (HbA1c) and risk of depression, while improving cognitive function.^17^ However, we^6^^,^^9^ and others^5^ have demonstrated that few MDs actually prescribe exercise to their patients. The primary barrier to prescription has been attributed in part, to a lack of available education^21^ which contributes to their inadequate knowledge and confidence. We previously observed no differences in the frequency of MDs providing exercise prescriptions in those who reported *some* previous training versus those who did not have any training on PAE, in a cross-sectional study of MDs in Nova Scotia.^6^ In that study, some training was loosely and broadly defined from attendance at a single symposium session on PAE, to attendance at workshop or taking a course. As such, there is a need for *effective* training opportunities that are pertinent and applicable to the clinical situation and can result in a greater inclusion of PAE content in more provider-patient appointments. Previous literature evaluating the impact of a single workshop-based educational training session observed favorable improvements in MDs physical activity counselling and exercise prescription knowledge, confidence and frequency of inclusion with their patients.^10^^,^^14^^,^^15^ A potential criticism of previous educational workshop-based studies is that they may have primarily included MDs who have moderate-high confidence in their ability to include PAE content with their patients. Specifically, the average initial confidence of workshop attendees was 51% (out of 100%) in our previous study^10^ and 6.2 (out of 10) in the study by Windt and colleagues.^15^ While Arciniegas Calle et al.^14^ did not specifically measure confidence in their study of Latin-American MDs, 73% of their sample reported assessing patient’s physical activity levels and providing counselling ‘often’ or ‘always’. The present study adds to the current literature by investigating ‘if’ and ‘how’ educational training alters clinical practice in MDs of varying initial confidence, which is important because many of these training opportunities are optimally targeted towards physicians who are not confident. As such, we were able to demonstrate that individuals with low initial confidence benefitted the most from this training; confidence increased by ~40% and the proportion of patients they assess PAE readiness, recommend PAE or prescribe exercise greatly increased. Therefore, a single interactive workshop has the capacity to produce significant short-term changes to practice among MDs who initially feel uncomfortable with their ability to provide physical activity and prescribe exercise to their patients (i.e., target primary care providers). The results of our study may be used to inform other researchers or healthcare providers in that medical education opportunities should consider the initial capabilities of the sample of interest when designing and evaluating medical education interventions. Clearly the magnitude of improvements in PAE perceptions and practices were influenced by their initial confidence with the low-confidence group increasing their confidence to a level reflective of the high-confidence group at follow-up (20-30% to 60-70%), whereas the high-confidence group demonstrated less of an improvement, but still favorable changes nonetheless (70-80% to 80-90%). Furthermore, medical educators may utilize the learning objectives of this workshop intervention and should consider additional recruitment strategies to target MDs who are not yet incorporating PAE content with their patients when designing educational training for primary care providers.

All three groups clearly demonstrated a greater confidence and provided written exercise prescription to more of their patients, more frequently following the educational workshop intervention. While all groups increased their awareness of guidance/resources and their perception of patients’ preferences for medication management, the low-confidence and medium-confidence groups still reported lack of time as an impactful barrier (2.3-2.4 out of 4), whereas, the high-confidence group reduced this barrier. It is likely that MDs need a higher level of confidence and comfort before they execute the skills of physical activity counselling and exercise prescription in a time efficient manner. Physicians in the higher confidence cohort may have already felt a solid level of competence in the more basic skills of assessing patient PAE readiness and making PAE referral when appropriate, therefore they did not show an improvement that met statistical significance in these areas following training. This difference may be clinically significant as *any improvement* in the area of PAE counselling and prescription practices of physicians are important in the context of health, chronic disease and assisting our physically inactive Canadian population, becoming more active. These improvements are particularly important since we know that patients who receive brief physical activity advice or written exercise prescriptions from their MDs increase their physical activity levels, as reviewed elsewhere.^17^^,^^18^ The other skills which did show a statistically significant improvement among the higher confidence group, generally require a greater level of personal knowledge and command of PAE, specifically, providing patient-centred individualized PAE information, answering specific questions relating to PAE asked by individual patients, and providing advice for special PAE considerations, such as patients with pre-existing chronic disease. Time-effective strategies or tools such as brief counselling,^18^ the physical activity vital sign,^22^ EIMC prescription pad,^23^ can be employed, but may require greater confidence to include in many appointments.

Importantly, the impact of perceived personal knowledge as a barrier greatly decreased (~25%) in the low- and medium-confidence groups, which then became similar in impact rating to that in the high-confidence group. It is likely that the increased confidence, personal knowledge and awareness of resources are responsible for the greater proportion of MDs with low confidence providing more their patients with written exercise prescriptions following the workshop. By perceiving their patient’s as more receptive to PAE content and/or time as less of a barrier, the medium- and high-confidence groups reported prescribing exercise to *even more* patients. Such findings demonstrate the importance of considering MDs initial confidence when evaluating the impact of continuing medical education opportunities and that great strides in PAE promotion may be conferred via a single training session.

At baseline, the low-confidence group perceived a lack of exercise education in medical training as a much more impactful barrier than those in the medium- and higher-confidence groups. These results are consistent with previous literature observing that Canadian medical residents (or trainees) perceive their current PAE training as inadequate.^24^ Despite being very effective, we acknowledge that interactive workshop-based training may be limited in reach with challenging logistics of reaching practicing physicians in the community *en masse*. However, this study does show efficacy with a simple educational workshop intervention. Our data highlight the need for more PAE training throughout the medical training continuum, from medical students through to practicing physicians since regardless of pre-workshop knowledge and confidence, all participating physicians benefitted from the training. The EIMC workshop learning objectives and main outcome measures which include providing; effective brief physical activity counselling, aerobic and resistance exercise prescriptions, motivational interviewing and utilizing the physical activity vital sign in patient encounters, may be useful in informing medical school curriculum development in PAE.

In addition to a greater emphasis in all stages of medical education, other strategies also need to be considered to support PAE counseling in regular clinical practice, such as continuing medical education opportunities,^21^ upstream changes (i.e. billing structure, physical activity vital sign in electronic medical record,^22^ etc.), availability of community exercise programs and resources, as well as collaboration between physicians and qualified exercise professionals^25^ to support patients when needed. While there are numerous personal reasons why some patients do not achieve the recommended levels of physical activity, greater medical education and the mentioned additional strategies would further support global, national and local efforts of reducing chronic disease by targeting the proportion of Canadians who are insufficiently active.^2^^,^^26^ Previous work has established that brief physical activity counselling is a resource and cost-effective means to promote physical activity through primary care.^27^ While a multi-factorial approach would likely result in the greatest change in the populations physical activity levels, our study shows the effectiveness of educational training on MDs with low confidence who initially were not discussing PAE during most patient appointments. We should expect that the inclusion of more PAE content in patient appointments will translate into a greater proportion of their patients meeting recommended physical activity levels.

This study is limited by a lack of a control group. We believe it is unlikely that MDs self-reported confidence and practices would change with repeated measures in a non-intervention group, particularly to the magnitude observed in the present study. Nevertheless, further research is needed. As with all survey studies investigating providers’ practices, participants may have responded in a socially desirable way, reporting higher confidence and rates of prescription. This potential limitation is believed to be minor as the social desirability bias is likely consistent at baseline and 3-month follow-up for each group and between groups. We acknowledge that our study did not evaluate whether the type of PAE recommendations or written exercise prescriptions (i.e., generic prescription vs. personalized prescription) were different between groups or in response to the training workshop. In addition to the frequency of PAE recommendations and written exercise prescriptions increasing, it may be possible that the quality of such recommendations or prescriptions also improved. The groups were broadly defined using confidence composite scores of < 40%, 40-60% and > 60%, respectively; however, the mean value for each category was 25±10%, 51±4% and 77±8% with all confidence variables significantly different between groups at baseline. Of relevance, the sample size of each group (*n =* 21; *n =* 19; *n =* 23) is reflective of another study investigating the impact of a training workshop on Canadian MDs with one-month follow-up (*n =* 25).^15^

### Conclusion

Our findings highlight that Canadian MDs who were and were not initially confident in their ability to perform PAE-related practices, reported favorable improvements in exercise prescription practices, PAE confidence and the perceived impact of many barriers, after attending the EIMC PAE training workshop. While all groups demonstrated favorable changes, how they achieved such improvements appear to be dependent upon their initial confidence. The target group of MDs (i.e. low-confidence) developed greater personal knowledge, awareness of guidance/resources for those with chronic disease and their perception of patient’s interest in lifestyle management, while those with high initial confidence found additional, time-effective ways to integrate PAE as part of their routine clinical care.
